# Anatomical Features of Intratemporal Course of Facial Nerve and its Variations

**DOI:** 10.7759/cureus.3085

**Published:** 2018-08-02

**Authors:** Raja Kalaiarasi, Avvaru Satya Kiran, Chellappa Vijayakumar, Ramakrishnan Venkataramanan, Manusrut Manusrut, Ravi Prabhu

**Affiliations:** 1 Otorhinolaryngology, Sri Lakshmi Narayana Institute of Medical Science, Puducherry, IND; 2 Otolaryngology, Rainbow Children's Hospital, Hyderabad, IND; 3 Surgery, Jawaharlal Institute of Postgraduate Medical Education and Research, Puducherry, IND; 4 Otolaryngology, Sri Lakshmi Narayana Institute of Medical Science, Pondicherry, IND; 5 Otolaryngology, ESIC Medical College, Hyderabad , IND; 6 General Surgery, Sri Lakshmi Narayana Institute of Medical Science, Pondicherry, IND

**Keywords:** temporal bones, facial nerve, mastoid, mastoidectomy, chorda tympani nerves

## Abstract

Introduction

Facial nerve has the longest and complex course in its bony canal. The anatomical variations make the nerve prone to injury during mastoid surgeries. Having a thorough anatomical knowledge and its variations is must for the surgeons to avoid injury to this vital structure and for the safe surgery. The objective of the study was to describe the anatomical variations of intratemporal portion of facial nerve.

Materials and methods

The study was conducted in the department of otorhinolaryngology in the temporal bone dissection laboratory of a tertiary health care centre. Fifty wet temporal bones were dissected by the same team of surgeons for the duration of one year to study the various anomalies of the intratemporal course of the facial nerve and its relations with the other important middle ear structures.

Results

The mean length of the labyrinth, tympanic and mastoid segment of the facial nerve was 4.1 mm (±0.6 mm), 9.34 mm (±1.12 mm) and 12.8 mm (±1.8 mm), respectively. The mean distance between the horizontal segment and posterior edge of the oval window was 3.1 mm (±1.03 mm). Dehiscence in the tympanic segment was observed in three temporal bones (6%). Two (4%) specimens had bifurcation of the mastoid segment of the facial nerve. In all dissected temporal bones, the chorda tympani travelled in an ascending path.

Conclusion

The most common site for facial nerve anomaly is the tympanic portion. Anomalous conditions that can place the nerve at risk of being injured by the surgeons are highlighted here.

## Introduction

Facial nerve is the important cranial nerve for the otolaryngologists which is known for its complex anatomy. Injury to the nerve results in facial disfigurement, dryness of the mouth and emotional disturbances in patients. The reported rate of iatrogenic injury to the facial nerve in primary tympanomastoidectomy surgeries was 0.6% to 3.7%. The risk is doubled in revision surgeries to 4% to 10% [1,2]. Though facial nerve monitors are being commonly employed in mastoid surgeries, it is still not accessible in many institutions in developing countries. Studying the anatomy of facial nerve through temporal bone dissection helps to avoid inadvertent injury to the nerve. Temporal bone dissection also helps to gain hand-eye co-ordination skills to excel in ear microsurgeries. The facial nerve has a pretty predictable course within the temporal bone so accidental injuries are rare in trained hands. Challenges arise when there are dehiscences in the bony canal and variations in the anatomy. Otolaryngologists should have a thorough knowledge of the normal anatomy and its variations to avoid injury to the nerve.

Fifty temporal bones harvested from cadaver’s skulls were dissected under the microscope to study the morphological features of the various segments of facial nerve and its relationship with other vital middle ear structures. The dissected temporal bones were studied for variations in the course of the facial nerve.

## Materials and methods

**Figure 1:**
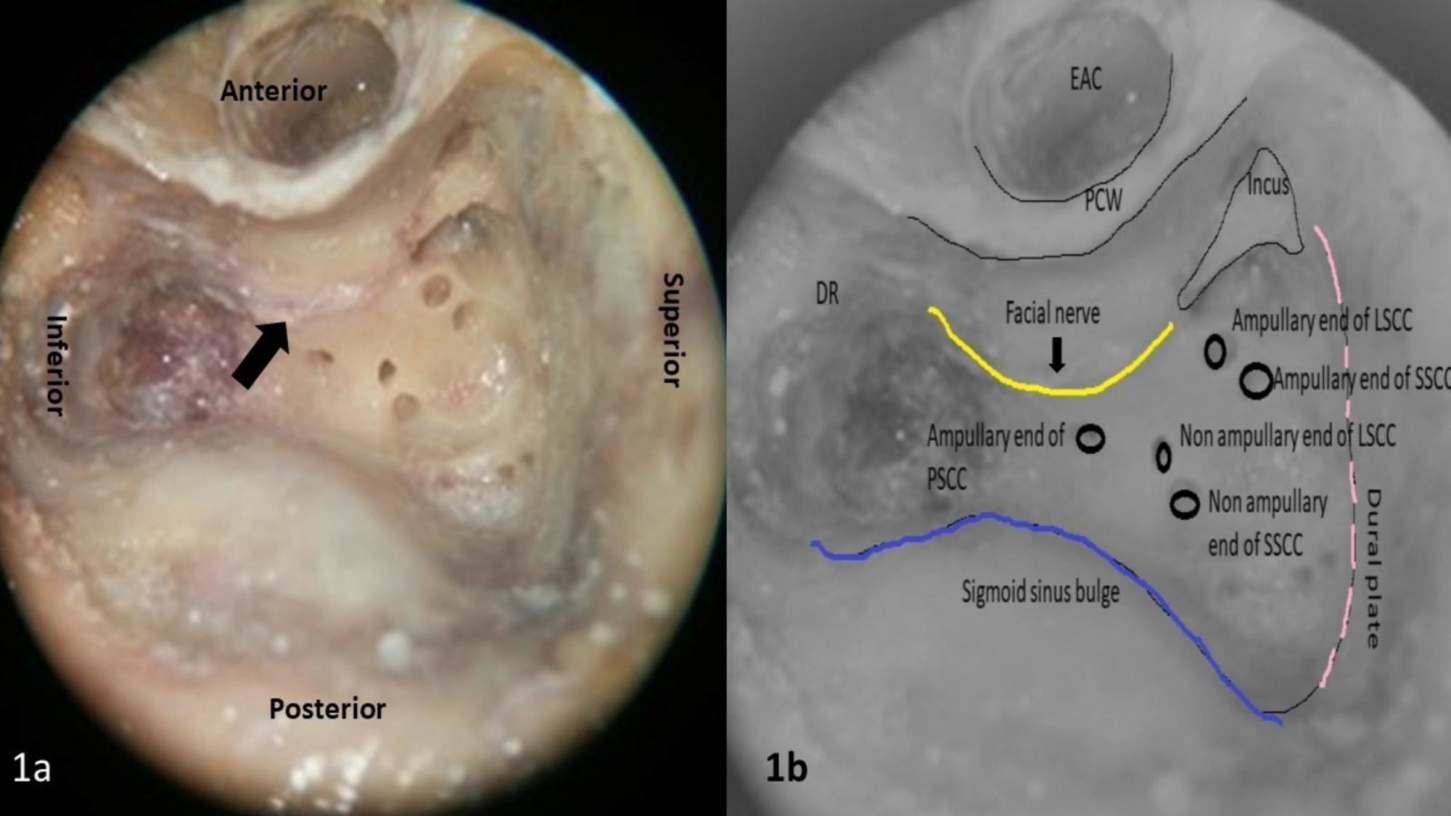
1a). Left temporal bone showing cortical mastoidectomy with labyrinthectomy showing facial nerve (black arrow) in the deepest part of the posterior canal wall. 1b). Illustrative picture showing structures in dissected temporal bone (yellow line: facial nerve, blue line: sigmoid sinus bulge and pink dotted line: dural or tegmen plate). DR: Digastric ridge; EAC: External auditory canal; PCW: Posterior canal wall; LSCC: Lateral semicircular canal; PSCC: Posterior semicircular canal; SSCC: Superior semicircular canal.

Dissection of 50 wet temporal bones preserved in 10% formalin was carried out over the one-year duration in the department of otorhinolaryngology temporal bone research laboratory in a tertiary health care centre. Dissection of the temporal bone was carried out with an operating microscope, using cutting and diamond burs of various sizes with continuous irrigation. In all bones, first cortical mastoidectomy was performed delineating dural plate superiorly, sinus plate posteriorly with exposure of sinodural angle, lateral semicircular canal in the floor and digastric ridge inferiorly (Figure 1).

**Figure 2:**
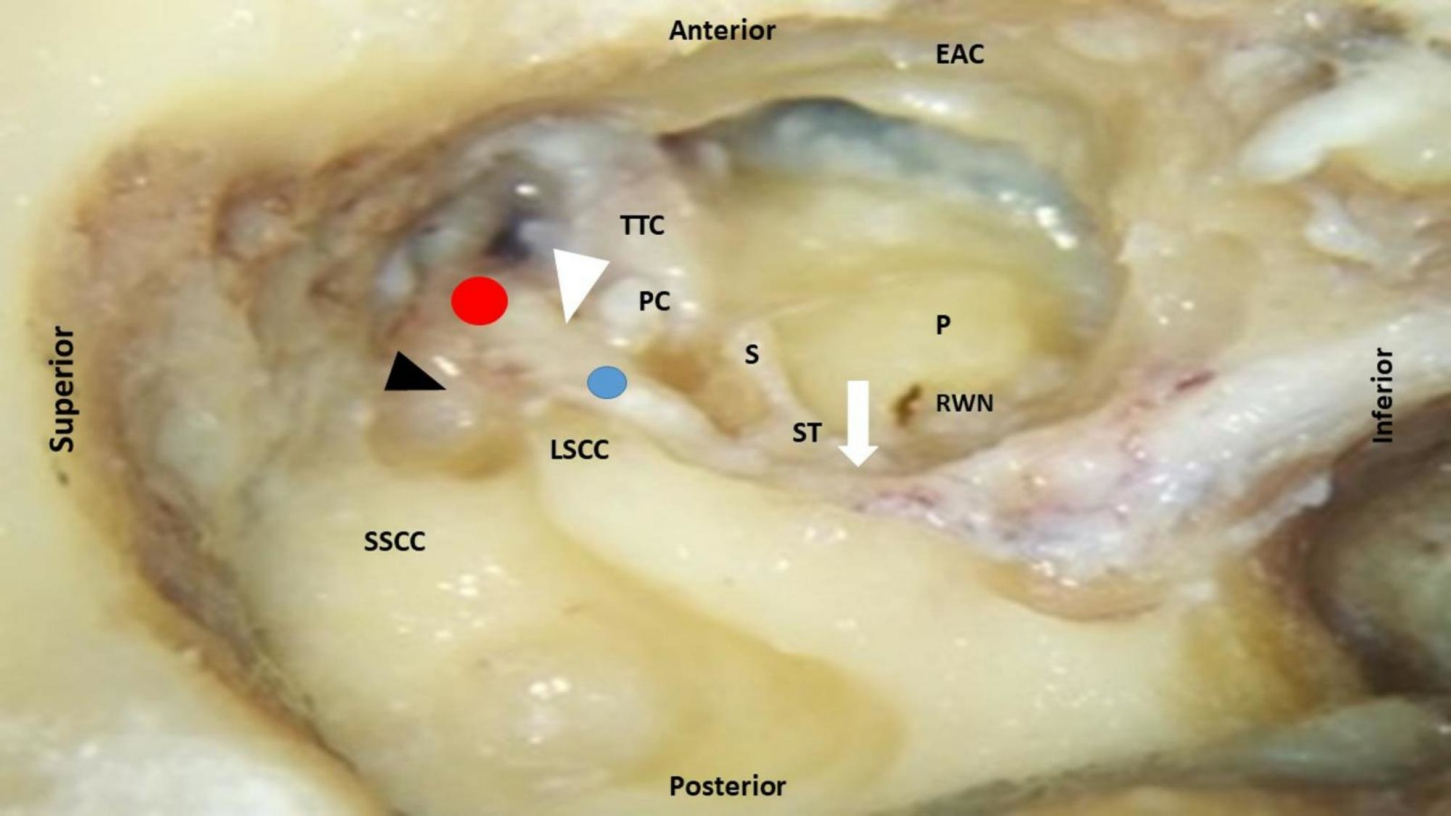
Right temporal bone showing labyrinthine (white arrow head), tympanic (black arrow head) and mastoid segment (white arrow) of the facial nerve. Red dot indicates first genu or geniculate ganglion and blue dot indicates second genu. EAC: External auditory canal (anterior wall); LSCC: Lateral semicircular canal; P: Promontory; PC: Processus cochleariformis; PSCC: Posterior semicircular canal; RWN: Round window niche; S: Stapes; ST: Stapedius tendon; TTC: Tensor tympani canal; SSCC: Superior semicircular canal.

After performing posterior tympanotomy procedure, the origin of chorda tympani nerve was noted. Anterior and posterior buttresses were removed and facial ridge was lowered. The facial nerve was delineated and its relationship with other middle ear structures was studied segment wise (Figure 2).

The lengths of labyrinthine segment, tympanic segment, and mastoid segment of the facial nerve were measured and their anatomical variations if any were studied and documented. Labyrinthine segment was measured from the fundus of the internal auditory canal to the geniculate ganglion. Geniculate ganglion was identified after removing the supralabyrinthine cells.

Tympanic segment or the horizontal segment of the facial nerve was measured from geniculate ganglion to second genu which is usually situated medial and inferior to lateral semicircular canal. Here it passes posteriorly and laterally along the medial wall of the middle ear. Mastoid or vertical segment was measured from second genu to stylomastoid foramen where the nerve exits from the temporal bone. Measurements were done using Vernier caliper, steel wires, dividers and protractor.

## Results

**Figure 3:**
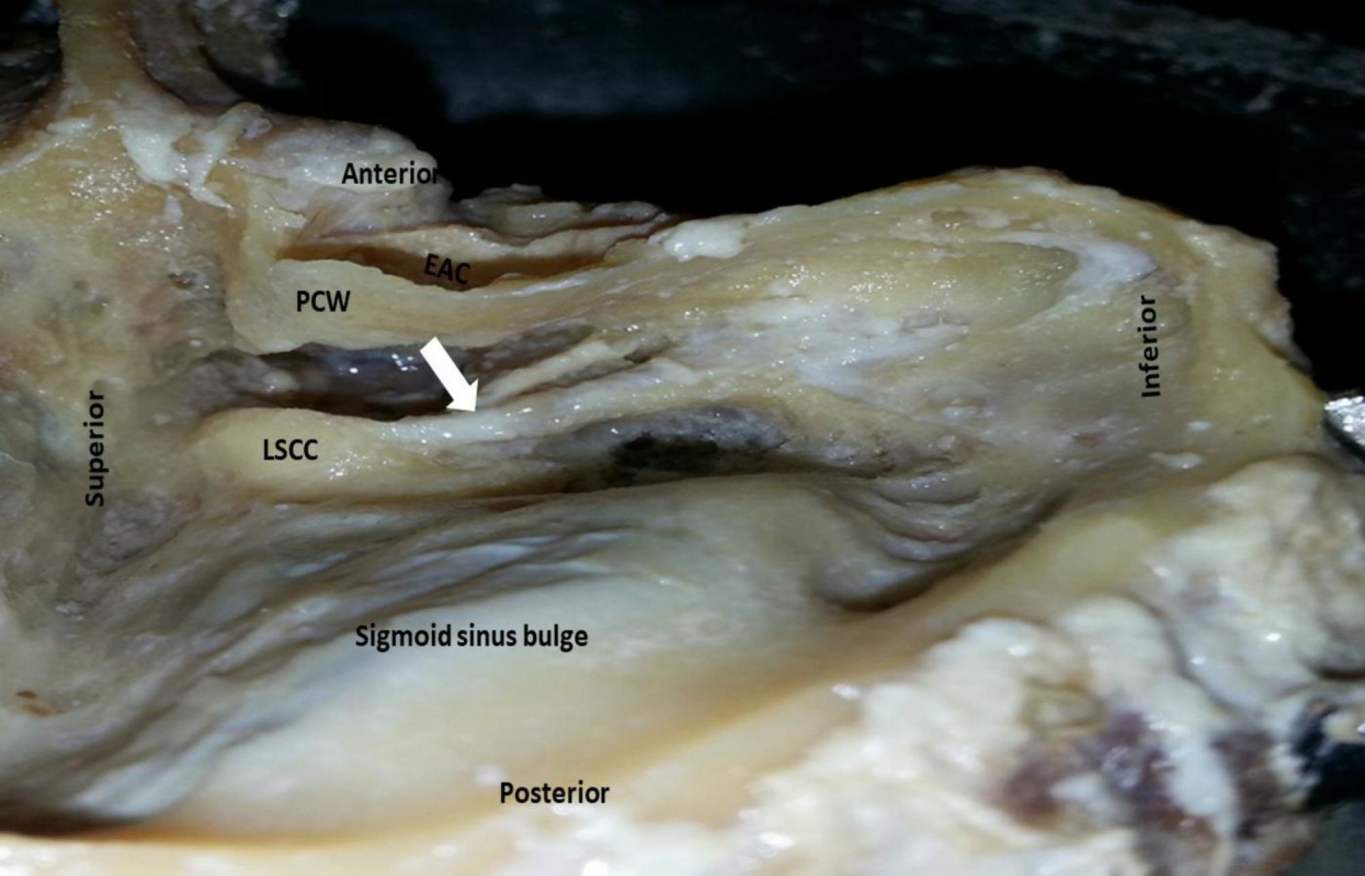
Right temporal bone showing bifurcation of the mastoid segment of facial nerve (white arrow). EAC: External auditory canal; PCW: Posterior canal wall; LSCC: Lateral semicircular canal.

Out of 50 wet temporal bones dissected, 46 bones were well pneumatized and four bones showed diploeic type of pneumatisation. Korner's septum was identified in four (8%) bones. The mean length of the labyrinth segment was 4.1 mm (±0.6 mm). The geniculate fossa contains geniculate ganglion from which greater petrosal nerve exits and travels anteriorly. There was no dehiscence noted at geniculate ganglion level. The geniculate ganglion is related medial and superior to the processus cochleariformis, the distance varied from 1.5 mm to 3.2 mm. The facial canal takes a turn and continues in the medial wall of middle ear as tympanic segment. The mean length of tympanic portion of facial nerve was 9.34 mm (±1.12 mm). In all temporal bones, the nerve passes behind the processus cochleariformis which is a landmark for the beginning of the horizontal segment. The facial nerve was posterosuperior to promontory and oval window and the nerve passed below lateral semicircular canal in all specimens. The distance between the beginning of horizontal segment and processus cochleariformis varied between 0.30 mm and 1.21 mm. The distance between the lateral semicircular canal and second genu was 1.9 mm (±0.52 mm). The mean distance between the horizontal segment and posterior edge of the oval window was 3.1 mm (±1.03 mm). Dehiscence in the tympanic segment was observed in three temporal bones (6%). The mean dehiscence was approximately 2 x 2 mm in size with smooth edges involving the lateral aspect of the facial canal and intact facial nerve sheath without any prolapse of the nerve. The tympanic segment continued as mastoid segment down vertically in the anterior wall of mastoid part of the temporal bone. The mean length of mastoid segment was 12.8 mm (±1.8 mm). The mastoid segment of the nerve was medial to tympanomastoid suture line. The facial nerve bony canal in the mastoid segment was intact in all 50 dissected specimens. Two (4%) specimens had bifurcation of the mastoid segment of the facial nerve (Figure 3).

The mean depth of second genu from the cortex was 19.12 mm (±1.8 mm). The distance between the origin of chorda tympani and stylomastoid foramen varied from 2 to 6 mm with a mean of 4.4 mm. In all dissected temporal bones, the chorda tympani travelled in an ascending path.

## Discussion

In the temporal bone, facial nerve might show various anomalies in its natural course. Anatomical variations of the facial nerve in the temporal bone are best appreciated by studying its embryological development. By the third week of gestation, facioacoustic neural crest cells appear from which the future facial nerve develops. By the fourth week of gestation, facial and acoustic portion becomes more distinct. The facial part of facioacoustic neural crest becomes narrow cell column which extends to otic placode located on the upper part of the second pharyngeal arch. Geniculate ganglion appears by the fifth week. At the same time, the proximal segment of primordium becomes more fibrous and less cellular. The distal segment separates into two branches of which one courses into the mesenchyme of the second pharyngeal arch forming the future facial nerve main trunk. The other branch crosses the first branchial arch which becomes chorda tympani. The facial motor nuclei are recognized by the end of fifth week of gestation.

By the seventh week, geniculate ganglion becomes more defined and nervus intermedius arises from the ganglion. The intratemporal portion of the nerve forms before extratemporal part and it courses from brain to second branchial arch. By the seventh week, as the tubotympanic recess becomes more prominent, the horizontal segment of facial nerve is apparent. By the eighth week of gestation, the membranous labyrinth reaches its adult framework thereby facial nerve establishes its relationships with other structures of the middle ear. The relationship of the facial nerve in the temporal bone is determined by embryological closure of the otic capsule sulcus to form fallopian canal. By the fifth month of gestation, the bone begins to cover the nerve but the facial nerve is not completely enclosed by bone even at birth [3]. Postnatally, the ossification process continues [4]. This is the reason why children are affected by facial nerve palsy secondary to acute otitis media due to higher incidence of dehiscent facial canal in the middle ear [5].

In the present study, the lengths of labyrinthine, tympanic and mastoid segment varied from 3.5–4.7 mm, 8.22–10.46 mm, 14.6–11 mm respectively which were in accordance with findings as observed by other authors [6-12]. In all dissected temporal bones, no major anatomical variations were observed in the course of labyrinthine segment and tympanic segment. Similar findings were observed by many authors [9-12]. Geniculate ganglion is located anteromedial and superior to processus cochleariformis. In our study, the distance between geniculate ganglion and processus cochleariformis was 1.5 mm to 3.2 mm. Maru et al. dissected 35 temporal bones to study the relationship of facial nerve with other structures. They found that geniculate ganglion was medial and superior to processus cochleariformis in 60% of cases and adjacent to it in 40% of cases [12]. The processus cochleariformis is a constant and safe landmark to facial nerve during mastoidectomy surgery. In our study, the distance between the beginning of horizontal segment and processus cochleariformis varied between 0.30 mm and 1.21 mm which is in accordance to the study by Maru et al. [12].

Incomplete closure of bony canal causes dehiscences which can put the nerve under danger during middle ear surgeries. Spector and Ge described dehiscences are normal anatomical variations rather than congenital defects [13]. Bony dehiscences are defined as a non-pathological breach of fallopian canal continuity of approximately 0.4 mm or larger. Microdehiscences are small gaps of less than 0.4 mm in size in the bony cover which are filled with connective tissue. It was observed that 57% of the population have dehiscence of the facial canal and dehiscences of tympanic segment in the oval window region being the most common region accounting for 74%. In the same study, complete dehiscence of tympanic segment was observed in four (0.74%) out of 539 dissected temporal bones [14]. In our study, we also observed dehiscences of tympanic segment in three temporal bones (6%). Complete dehiscence of tympanic segment was not observed in any specimen. In contrary to our study, vertical segment of the fallopian canal dehiscence was observed in 9% of specimens [14].

The nerve may course with bifurcation, trifurcation or other aberrant pathways within the temporal bone. Bifurcation of the facial nerve is a rare variant with focal splitting of one or more segments [15,16]. The mastoid segment of the nerve runs in a line starting from fossa incudis where short process of incus lies to anterior end of the digastric ridge. In the present study, two specimens (4%) showed bifurcation of mastoid segment, one larger lateral segment branch emerging out through stylomastoid foramen and other smaller medial segment branch exiting from petrotympanic fissure. The chorda tympani branch emerging from the larger lateral segment. Anson et al. observed bifurcation of the nerve in three out of 500 temporal bones dissected randomly [11]. Surgeons should be aware of this extremely rare variation as facial nerve trunk can be damaged during surgical procedures like facial nerve decompression and parotid surgeries.

## Conclusions

In-depth knowledge of the anatomical features of the facial nerve in temporal bone is crucial for all otolaryngologists. Better understanding of this complex structure through temporal bone dissection will lead to safer ear surgery. Knowledge of the anomalies of facial nerve is useful in presurgical evaluation to avoid inadvertent nerve injury.
